# Wearable Multi-Channel Pulse Signal Acquisition System Based on Flexible MEMS Sensor Arrays with TSV Structure

**DOI:** 10.3390/biomimetics8020207

**Published:** 2023-05-18

**Authors:** Xiaoxiao Kang, Lin Huang, Yitao Zhang, Shichang Yun, Binbin Jiao, Xin Liu, Jun Zhang, Zhiqiang Li, Haiying Zhang

**Affiliations:** 1 Institute of Microelectronics of Chinese Academy of Sciences, Beijing 100029, China; 2 University of Chinese Academy of Sciences, Beijing 100049, China; 3 Beijing Key Laboratory for Next Generation RF Communication Chip Technology, Beijing 100029, China

**Keywords:** pulse wave, MEMS sensor, flexible arrays, TSV, wearable, Traditional Chinese Medicine, pulse width

## Abstract

Micro-electro-mechanical system (MEMS) pressure sensors play a significant role in pulse wave acquisition. However, existing MEMS pulse pressure sensors bound with a flexible substrate by gold wire are vulnerable to crush fractures, leading to sensor failure. Additionally, establishing an effective mapping between the array sensor signal and pulse width remains a challenge. To solve the above problems, we propose a 24-channel pulse signal acquisition system based on a novel MEMS pressure sensor with a through-silicon-via (TSV) structure, which connects directly to a flexible substrate without gold wire bonding. Firstly, based on the MEMS sensor, we designed a 24-channel pressure sensor flexible array to collect the pulse waves and static pressure. Secondly, we developed a customized pulse preprocessing chip to process the signals. Finally, we built an algorithm to reconstruct the three-dimensional pulse wave from the array signal and calculate the pulse width. The experiments verify the high sensitivity and effectiveness of the sensor array. In particular, the measurement results of pulse width are highly positively correlated with those obtained via infrared images. The small-size sensor and custom-designed acquisition chip meet the needs of wearability and portability, meaning that it has significant research value and commercial prospects.

## 1. Introduction

Pulse diagnosis has been an indispensable aspect of Traditional Chinese Medicine (TCM) since ancient times, playing a crucial role in diagnosis and treatment. Studies have shown that pulse waves contain adequate information reflecting the state of the human heart and blood vessels [1,2]. This information can be utilized to predict and reflect a variety of cardiovascular disease states in clinical TCM practice, such as hypertension [3], coronary heart disease [4,5], arterial stiffness [6], vascular aging [7], and atrial fibrillation [8]. Given its non-invasive, convenient, and rapid diagnosis, pulse diagnosis has significant potential for application and development. As sensor and signal processing technologies continue to advance [9,10,11,12], the objective and digitalization of pulse diagnosis have become research hotspots.

The modern development of pulse diagnosis in TCM is limited by the heavy reliance on the experience of experts. However, existing TCM theories indicate that pulse diagnosis relies on a combination of previous experience and clinical practice. The fingertip-sensing components of pulse waves are decomposed into multiple characteristic dimensions, including pulse wave amplitude, width, frequency, etc. [13,14]. Human fingertips can essentially capture the three-dimensional (3D) spatial pulse information. To simulate the pulse diagnosis process of TCM, 3D pulse wave detection technology can be utilized [15,16]. The 3D information of the pulse mainly includes the coordinate information and intensity information of the sensed sensor unit. Therefore, the pulse wave sensor and processing method become crucial factors affecting the accuracy of pulse collection and analysis.

Various types of sensors have been utilized for pulse wave acquisition, including optical [17], piezoelectric sensors [5,18,19,20], ultrasonic [20,21], and pressure sensors [10,22]. Recently, some researchers have studied fiber optic sensors [23,24] and RF sensors [25]. However, these sensors ignore the external static pressure applied when collecting pulse waves, which is essential in TCM. There are also some studies that have directly prepared sensors on flexible substrates to collect pulse waves [26,27,28,29,30,31]. Commonly used materials include polyimide (PI) film materials, polydimethylsiloxane (PDMS), and polyethylene terephthalate alcohol esters (PET). Their good flexibility and stretchability show great potential in future wearable devices. Nevertheless, the consistency, repeatability, external impact resistance, and linearity of the flexible sensors are still much lower than silicon micro-electro-mechanical system (MEMS) piezoresistive sensors. MEMS pressure sensors are often employed in array pulse wave acquisition systems due to their small size and stable signal characteristics [32]. However, the current MEMS pressure sensor design connects the flexible substrate to the sensor using gold wire bonding, which can cause problems such as wire bond failure due to the compression and bending of the flexible substrate. This issue would affect the detection pressure range and service life of the sensor [15,16,33]. Developing a sensor that can maintain high accuracy and a large pressure detection range without the need for gold wire bonding is key to addressing this problem.

There are also some challenges in establishing an efficient mapping between the array sensor signal and the pulse width. In TCM, the pulse wave width is closely related to blood pressure and blood flow, which is indispensable in the pulse signal acquisition system. The width of the pulse wave does not accurately represent the diameter of the radial artery but rather the activity of the radial artery and its surrounding tissues during the pulse [34]. Under the action of external static forces, the amplitude and width of the pulse wave also change. Therefore, the acquisition system should be able to synchronize the amplitude, static pressure, and width of the pulse wave. Meanwhile, single-point pulse wave sensors can only detect pulse waves at one site of the wrist, and the one-dimensional intensity signal obtained cannot represent the pulse information of the entire wrist region. At present, pulse wave sensing arrays have been proposed, and two arrays have been shown in our previous work to obtain pulse wave signals; however, due to the limitations of the sensor array size and the effective information connection between the pulse sensor signals and 3D pulse wave, the pulse width cannot be calculated. In the existing research [14,15,16], there are also methods of using arrays to measure pulse width, but there are problems of large sensor size, manual positioning, and insufficient accuracy. More complex signal processing circuits limit their application in wearable and portable scenarios [35,36]. There is an urgent need to design a small-size pulse wave sensor flexible array [37,38,39] and a signal processing circuit [40].

To resolve the aforementioned challenges, we propose a wearable multichannel pulse wave acquisition system based on a novel pulse wave MEMS pressure sensor. The main contributions of this paper are as follows:

1. A novel MEMS pulse pressure sensor that features a through-silicon-via (TSV) structure is proposed in this paper. Notably, the TSV design allows for direct connection to the flexible substrate, eliminating the common issue of sensor failure due to gold wire breaking, which is typically associated with conventional MEMS pressure sensors utilizing gold wire bonding.
2. Based on the MEMS sensor, a 24-channel pulse sensor flexible array is engineered, which can completely cover the sensitive area of the radial artery of the wrist and collect pulse wave and static pressure simultaneously.
3. The array is equipped with a self-customized multichannel pulse preprocessing chip, which accomplishes signal amplification and converts the pulse analog signal into a digital signal.
4. An algorithm is developed to reconstruct the 3D pulse wave from the pulse wave array signal and calculate the pulse width. The pulse width calculation result is highly positively correlated with those obtained via infrared images.
5. The experimental results and analysis verified the system’s efficacy and repeatability.

The rest of this paper is organized as follows: In the Materials and Methods section, we elaborated on the system composition, MEMS pressure sensor, flexible MEMS pressure sensor array, multi-channel pulse wave preprocessing chip, and 3D pulse wave and pulse width measurement method. In the experimental results and discussion section, we tested the performance of the MEMS pressure sensor and the performance of the sensor array and carried out experiments on pulse wave acquisition and pulse width measurement to verify the effectiveness of the sensor and system. In the conclusions section, we summarized the design and performance of the system.

## 2. Materials and Methods

### 2.1. System Overview

**Figure 1:**
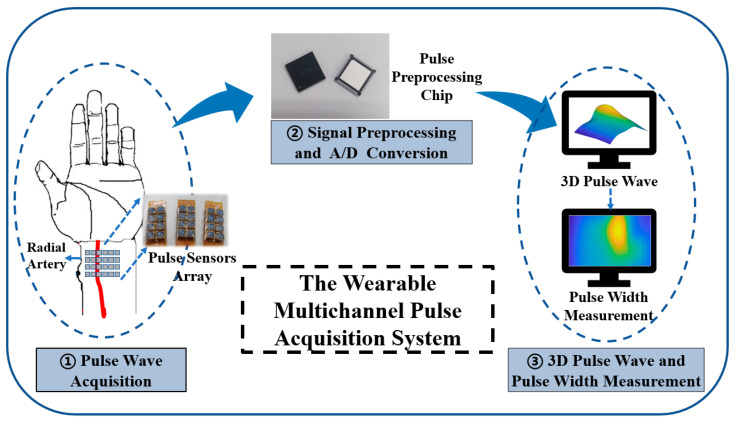
The schematic diagram of our proposed wearable multichannel pulse wave acquisition system.

Our proposed wearable multichannel pulse wave acquisition system is comprised of three modules. The first module is a 24-channel flexible MEMS sensor array, which is worn on the human wrist to acquire wrist radial pulse wave signals. This array is based on a MEMS pressure sensor with a TSV structure, which can acquire pulse wave signals at continuously varying static pressures. The sensor array is fixed in a specially customized structural housing. The second module is a custom-designed pulse signal special processing circuit, which accomplishes preprocessing, including signal amplification, and converts the pulse analog signal into a digital signal. The third module is the 3D pulse wave and pulse width calculation algorithm module, which recovers the 3D pulse wave from the array signal and calculates the pulse width information. The schematic diagram of the wearable multichannel pulse wave acquisition system proposed is shown in Figure 1.

The flexible MEMS sensor array represents significant progress as it uses a TSV structure that allows a direct electrical connection to the flexible substrate through the silver paste, avoiding the problem of sensor failure after the gold wire breaks. After collecting pulse wave signals, the preprocessing chip accomplishes signal amplification, converts the pulse analog signal into a digital signal, and sends the signals to a smartphone or PC via Bluetooth. The algorithm processes the pulse wave array signal into a 3D pulse wave signal, which is used for analyzing and calculating pulse width.

### 2.2. MEMS Pressure Sensor with TSV Structure

**Figure 2:**
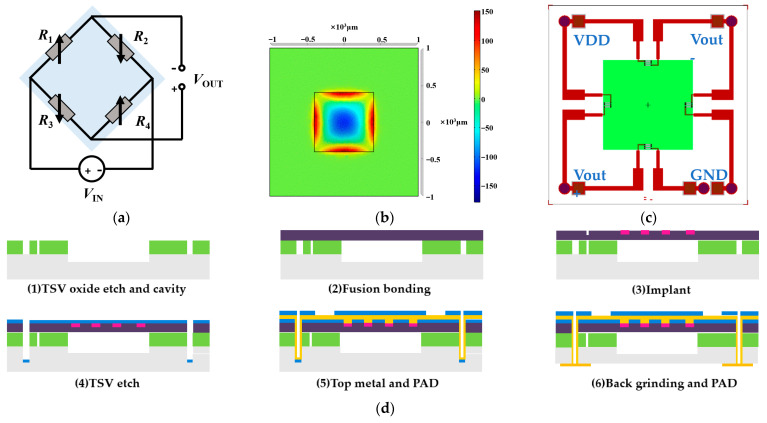

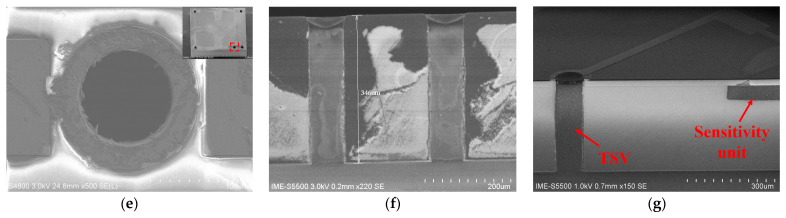
Design process of the MEMS pressure sensor with a TSV structure. (**a**) Schematic diagram of the circuit structure of the pressure sensor. (**b**) The pressure-strain effect of the film at 120 KPa. (**c**) Schematic diagram of the structure of a MEMS pressure sensor with a TSV structure. (**d**) Schematic diagram of the MEMS sensor production process. (**e**) Schematic diagram of the front side of the scanning electron microscope (X-SEM) of the TSV structure. (**f**) Schematic diagram of the TSV structure section X-SEM. (**g**) X-SEM image of TSV and sensitive unit.

The proposed flexible sensor array employs MEMS piezoresistive pressure sensors as the fundamental building block. In contrast to existing MEMS pulse pressure sensors and flexible substrates with gold wire bonding, our design features a TSV structure to allow for the reverse output of the electrical signal. Furthermore, the back of the sensor can be directly connected to the flexible substrate. The working principle of the sensor is to use the piezoresistive effect of silicon and use four force-sensitive resistors to form a Wheatstone bridge. The schematic diagram of the sensor circuit structure is shown in Figure 2a. The initial resistance value of $R_{1}$ = $R_{2}$ = $R_{3}$ = $R_{4}$ is balanced with zero input, resulting in zero output. When external pressure is exerted on the sensor’s elastic diaphragm, a voltage difference arises on both sides of the film, causing the resistance of the four force-sensitive resistors to change. As a result, the Wheatstone bridge becomes unbalanced, generating a voltage output:

(1) $$V_{OUT}=V_{IN}\frac{\left(R_{1}+{∆R}_{1}\right)\left(R_{3}+{∆R}_{3}\right)-\left(R_{2}+{∆R}_{2}\right)\left(R_{4}+{∆R}_{4}\right)}{\left(R_{1}+{∆R}_{1}+R_{2}+{∆R}_{2}\right)\left(R_{3}+{∆R}_{3}+R_{4}+{∆R}_{4}\right)}$$

where $V_{IN}$ is the power supply voltage, $V_{OUT}$ is the output voltage, and ${∆R}_{1}$, ${∆R}_{2}$, ${∆R}_{3}$, and ${∆R}_{4}$ are the resistance changes of the bridge arm caused by pressure.

The MEMS pressure sensor is designed with a full scale of 120 KPa, which can fully meet the collection requirements of pulse wave pressure and static pressure applied to the sensor. The structural design of the sensor includes an elastic diaphragm design and a varistor design. The elastic diaphragm is the sensitive element of the sensor, and the force-sensitive resistor is prepared on the elastic diaphragm. Considering its miniaturization and easy mass production, the shape of the elastic diaphragm was chosen as a square elastic diaphragm. For a square silicon diaphragm, the effective side length l and diaphragm thickness h of the silicon diaphragm should satisfy as follows:

(2) $$\frac{l}{h}\leq \sqrt{\frac{4\sigma }{3p}}$$

where p is the maximum pressure of the sensor and σ is the allowable stress value of the film structure. To ensure a good linear relationship between the diaphragm stress and the external pressure, the deformation of the working diaphragm of the sensor must meet the small deflection theory; the maximum deflection $\omega_{max}$ of the elastic membrane deformation of the MEMS piezoresistive pressure sensor satisfies as follows:

(3) $$\omega_{max}=0.0151\frac{pl^{4}}{Eh^{3}}(1-\mu^{2})\leq 20\%h$$

where E is the elastic modulus of single-crystal silicon and μ is Poisson’s ratio. We optimized the film structure of the sensor by simulating the parameters such as film stress and strain. The pressure-strain effect of the film at 120 KPa is shown in Figure 2b. Therefore, according to the size of the sensor chip, the thickness of the elastic film is 10 µm. For the varistor design, the shape of the resistor is shown in Figure 2c, and the optimal placement of the varistor strips can be clearly obtained from Figure 2b to maximize the use of the piezoresistive effect of the silicon material. The schematic structure of the absolute pressure sensor with a TSV structure is shown in Figure 2c, with a single device size of 2 mm × 2 mm and a total of five TSV structures in each device, realizing the direct interconnection of the back metal pad and the front varistor strip structure.

The manufacturing process of the MEMS pressure sensor we developed is shown in Figure 2d. (1) TSV oxide etch and cavity: a blocking layer was deposited on the silicon substrate, the TSV blocking layer was etched, and a pit was etched on the silicon substrate as a sensor cavity. (2) Fusion bonding: the cavity-SOI process was used to realize the precise control of sensitive film thickness. A silicon oxide-strained film above the cavity with a film thickness of 10 µm via fusion bonding was prepared. The top silicon was thinned after bonding. (3) Ion implantation (boron doping) was used to prepare force-sensitive resistor strips. The ion implantation depth was 1 µm, and the aspect ratio was 15:1. (4) TSV etching: the filling material of TSV was tungsten, and the filling method was chemical vapor deposition (CVD). (5) Top metal and pad: the TSV structure and the leads and pads on the front side of the sensor were prepared. (6) Back mill and pad: the back was thinned and polished to the desired device thickness, and the leads and pads on the back of the sensor were prepared. The sensitive unit was designed in the center, and the TSVs were located in the four corners of the chip. The bottom solder ball realized the integration with the system board. We developed an 8-inch wafer tape-out for the MEMS pressure sensor. The front diagram of the scanning electron microscope (X-SEM) of the TSV structure is shown in Figure 2e. The schematic diagram of the TSV structure section X-SEM is shown in Figure 2f. The X-SEM image of TSV and the sensitive unit is shown in Figure 2g.

We carried out the short process development of key processes, including the bonding of silicon and silicon oxide with the pit structure, the development of the resistance strip’s resistance consistency, and the development of the TSV front- and back-side connection. Through the development of the doping process of the resistor strips, the resistance values of the four varistors of a single device can be optimally consistent. The I–V test results of 4 resistance strip resistance values of a single device were 4.06 KΩ, 4.112 KΩ, 4.064 KΩ, and 4.113 KΩ, with an average value of 4.087 KΩ. The TSV structure had a depth-to-width ratio of 4:1. After deep silicon etching and metal filling, the impedance of the TSV itself was 3.0359 Ω, and the effect of front- and back-side communication was well-realized.

The MEMS employs a structural approach that is integrated with the TSV process, effectively solving the force failure issue of the traditional piezoresistive absolute pressure sensors when receiving pulse signals. Through the optimization of design parameters, such as the structure of the piezoresistive strain film and varistor strip position, and precise control of the sensitive film thickness via the bonding process, we created a sensor with high sensitivity and precise performance. Section 3.1 presents the performance test method and verification results of the sensor.

### 2.3. Flexible MEMS Pressure Sensor Array

Utilizing the aforementioned MEMS pressure sensor with a TSV structure, we developed a pliant pressure sensor array specifically tailored for pulse wave acquisition. The flexible sensor array comprises 4 rows and 6 columns of MEMS sensors with a row spacing of 6.3 mm and a column spacing of 5.2 mm, where the distance is calculated as the distance from the center of one sensor to the center of another. The total size of the sensor array is 20.9 mm × 28 mm, which completely covers the radial pulse acquisition area of the wrist and is designed for wearing on the wrist.

**Figure 3:**
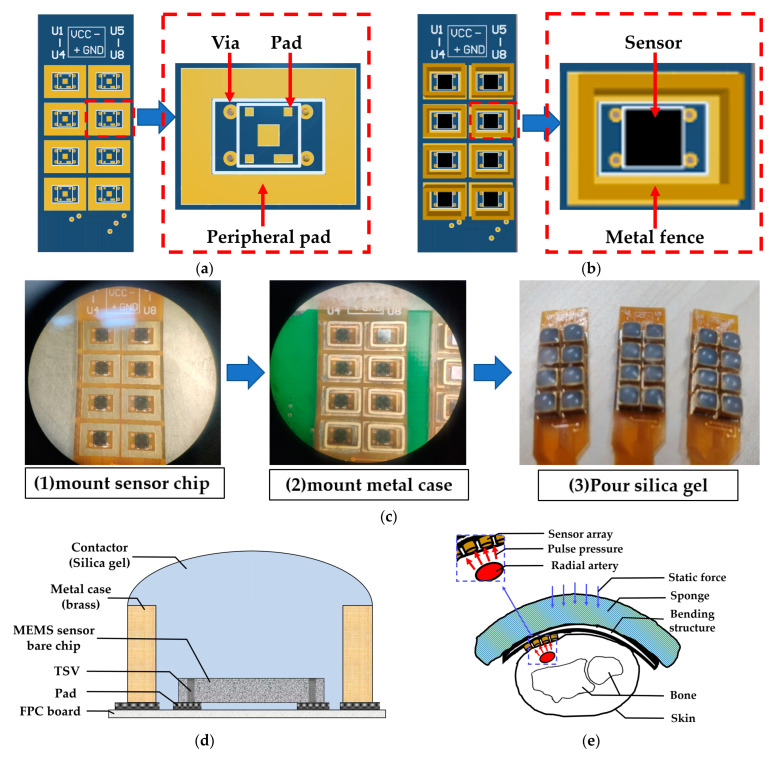
Flexible MEMS pressure sensor array. (**a**) Design diagram of flexible printed circuit board (FPC). (**b**) Package diagram of a single sensor. (**c**) Production process of packaged sensor. (**d**) Schematic diagram of package structure. (**e**) Application demonstration diagram of sensor array.

The production process of the packaged sensor is shown in Figure 3. A crucial aspect of designing and fabricating the sensor array is identifying an intermediate layer material that facilitates pulse pressure conduction and safeguards the sensor chip. Following experimentation and testing, we determined that liquid silicone is an ideal material for this intermediate layer as it has resilience, thermal stability, water resistance, gas permeability, and heat aging resistance. Firstly, we customized the design of the flexible printed circuit board (FPC), which features a design schematic as illustrated in Figure 3a,b, including FPC pads coated with silver paste that can be directly connected to the bottom surface of the sensor chip. Here, the back of the pad contains a glass-reinforced epoxy resin laminate that acts as reinforcement and support for the sensor chip. We then place the FPC board connected to the chip in a dryer at a temperature of 150 °C for 50 min to solidify the silver paste. Secondly, after completing the chip surface bonding, we applied solder paste to the peripheral pad and added a metal case, to contain the liquid silicone and for protection, before placing it in a dryer for drying. Thirdly, we inject an appropriate amount of liquid silicone into the fence formed by the metal case with a needle tube, and then placed the FPC plate in a ventilated area for about 2 to 3 h, allowing the silicone to solidify. Figure 3c depicts the production flow chart of the sensor array packaging, and a physical photo of the packaged sensor array is displayed in Figure 3c (3). Figure 3d showcases a cross-sectional diagram of a packaged sensor. The entire array of 24 sensors was produced in accordance with the aforementioned process. The sensor array is designed to cover the entire radial artery region of the wrist, ensuring that the sensor can accurately measure pulse waves.

With the sensor array prepared, we assembled the sensor to fix it on the wrist to stably collect pulse waves. Similar to our team’s previous research [16], we designed a curved housing structure for the sensor array, and the curvature of the housing was tailored to fit the characteristics of most people’s wrists, maintaining good contact between the sensor and human skin and allowing the user to feel comfortable when collecting pulse waves. The cross-section diagram of the sensor array module measuring the pulse wave of the human wrist is shown in Figure 3e, in which the red arrow represents the dynamic pulse wave pressure, the blue arrow represents the static pressure applied when collecting the pulse wave, and the pulse wave signal is detected through the sensor array.

### 2.4. Multichannel Pulse Preprocessing Chip

The pulse wave signals acquired by the flexible MEMS pressure sensor array described in the previous section are fed into our custom-designed multichannel pulse preprocessing chip. The flexible MEMS pressure sensor array consisted of 24 MEMS sensors that output 24 channels of pulse wave differential signals. The larger amplitude DC component corresponded to the applied static pressure, while the smaller amplitude AC component corresponded to the pulse wave pressure. We used a filter to extract the AC component from the output voltage signal of the sensor, i.e., a small dynamic pulse wave signal, and used a low-noise instrumentation amplifier to amplify it 100 times to obtain a signal with a higher signal-to-noise ratio. The DC component was amplified 10 times, and then a 1.6 V bias voltage was added to meet the sampling voltage range of the ADC (0–3.3 V).

**Figure 4:**
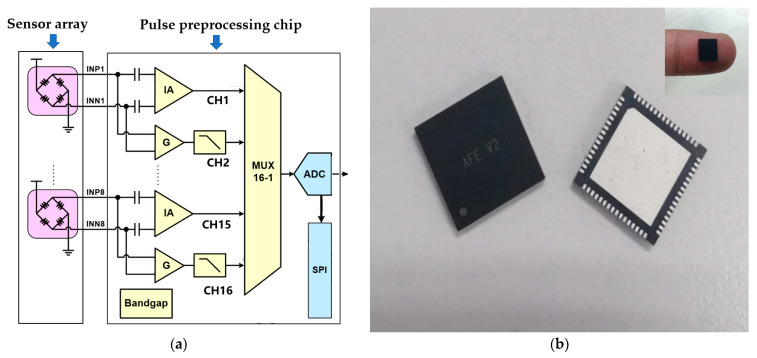
Multichannel pulse preprocessing chip. (**a**) The circuit architecture design diagram inside a single chip. Each chip completed a total of 8 channels of pulse wave signal processing, and a total of 3 chips completed the pulse wave processing of 24 channels. (**b**) Physical diagram of a single pulse preprocessing chip.

We employed three self-designed tape-out pulse preprocessing chips to complete processing and A/D conversion, and each chip realized the acquisition and processing of 8 channels of pulse wave signals. The circuit architecture diagram inside each chip is shown in Figure 4a. After completing the amplification, we used a multiplexer to convert the multichannel signal into a single signal. Each chip had an ADC to convert an analog signal into a digital signal, and the circuit sampling rate was 1 Msps with a resolution of 10 bits. The system sampling frequency was 256 Hz, which exceeded that of the pulse wave signal. The data packet formed by the sampled signal was ultimately conveyed to the PC through Bluetooth. The Bluetooth module used for wireless transmission was nRF52810, which supported the Bluetooth 5.0 low-power protocol and is a low-power Bluetooth chip; it had a 2.482 × 2.464 mm package, which is the smallest package of all nRF52 series. The maximum data transfer rate reached 2 Mbps. The sampling rate of the pulse wave signal was 256 Hz, and the data obtained by sampling each channel per second was 512 B. In addition to the custom data transfer protocol, the maximum amount of data transferred per second was 12.2 KB. The Bluetooth module meets the needs of wireless data transmission. During data acquisition, data was transmitted continuously.

To meet the needs of wearable mobile acquisition, the multichannel pulse preprocessing chip architecture proposed here adopted a low-power, miniaturized design that utilized the SMIC 0.18 μm logic and mix-signal CMOS process in its design, simulation, and tape-out. An internal, low-noise instrumentation amplifier was integrated to enhance weak electrical signals and nullify the DC offset voltage from the electrodes, thereby minimizing the 1/f noise. The chip was capable of supporting 8 parallel pulse wave channel input signals with an operating voltage of 5 V and a current consumption of 3.740 mA. Additionally, the single-chip power consumption was only 18.7 mW. Furthermore, the chip employed the Quad Flat No-leads (QFN) Package, resulting in a reduction in the system size. The physical diagram of the chip can be observed in Figure 4b, and the parameter table can be seen in Table 1.

### 2.5. 3D Pulse Wave and Pulse Width Measurement

After receiving the data packets of the pulse wave, the computer took out the signals of each channel in turn and then restored the signal to 24-channel dynamic pulse wave signals and 24-channel static pressure signals. We then processed and stored the pulse wave signal in the software program. We denoised the collected signal to filter out 50 Hz power frequency interference. Then, a median filter was employed to remove anomalies in the sampling process. Furthermore, an average filter and a low-pass filter with a cutoff frequency of 15 Hz were utilized to eliminate the interference caused by baseline drift.

**Figure 5:**
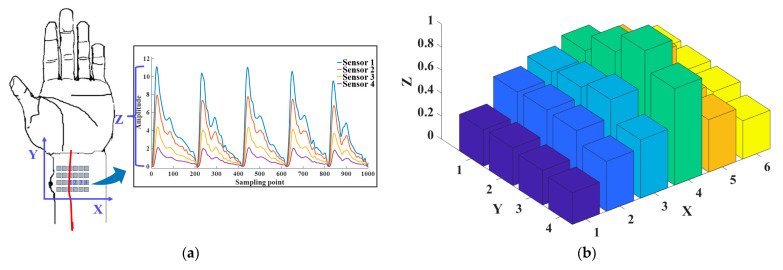

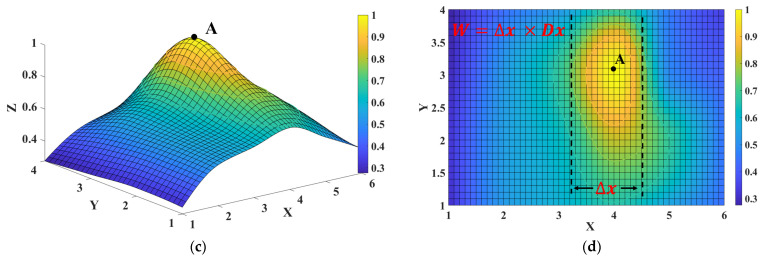
Three-dimensional (3D) pulse wave signal reconstruction and pulse width calculation. (**a**) Schematic diagram of the X-axis, Y-axis, and Z-axis of the 3D pulse wave. (**b**) Histogram of radial pulse wave signal intensity distribution of 24 channels. (**c**) The 3D pulse wave of the radial artery constructed from the 24-channel pulse signal via the bicubic interpolation method. (**d**) Schematic diagram of pulse width calculation using the formula W=∆x×Dx, where Dx is the row distance 4.3 mm and ∆x is the distance of the contour in the X-axis.

The 3D pulse wave was reconstructed after signal denoising was completed. The 3D pulse wave signal reflects the amplitude change of the pulse wave in the spatial position, which was more in line with the finger feeling of TCM doctors. As shown in Figure 5a, the pulse wave width direction is the X-axis, the pulse wavelength direction is the Y-axis, and the pulse wave amplitude acquired by each sensor in the array is the Z-axis. The array pulse wave signal is presented in the form of a 3D pulse wave histogram consisting of 24 points, as shown in Figure 5b. We normalized the pulse wave amplitude. The histogram shows the distribution of the pulse wave intensity and describes the intensity fluctuation of the wrist pulse wave signal in space.

To reconstruct the 3D pulse wave more accurately, we utilized a surface fitting method to convert the histogram into a smooth 3D pulse wave. This was achieved using bicubic interpolation, which concatenated the pulse wave intensities from each sensor and created a pulse wave intensity mapping surface for cubic interpolation in the X and Y directions. The smooth 3D pulse wave is shown in Figure 5c. The pulse intensity along the radial artery was stronger than that around, which was consistent with existing studies [4,14]. The 3D pulse wave was more in line with the pulse diagnosis finger feeling of a TCM doctor.

We calculated the pulse width using the processed 3D pulse wave signal. Firstly, for the 3D pulse wave signal, we located the peak point and marked it as point A. When the pulse wave amplitude at point A reached the peak value, we set the contour line according to the threshold value of the pulse wave amplitude. For a more intuitive display, we mapped the 3D pulse wave to a 2D plane, as shown in Figure 5d. The three contour lines in the figure, the top line, and the bottom line, respectively, represent the upper and lower thresholds of the pulse width of healthy people [34], and the middle line represents the pulse width of the collected pulse wave. The pulse wave width is calculated by the formula W=∆x×Dx, where Dx is the row distance 4.3 mm and ∆x is the distance of the contour in the X-axis.

## 3. Experimental Results and Discussion

### 3.1. MEMS Pressure Sensor Performance Test

**Figure 6:**
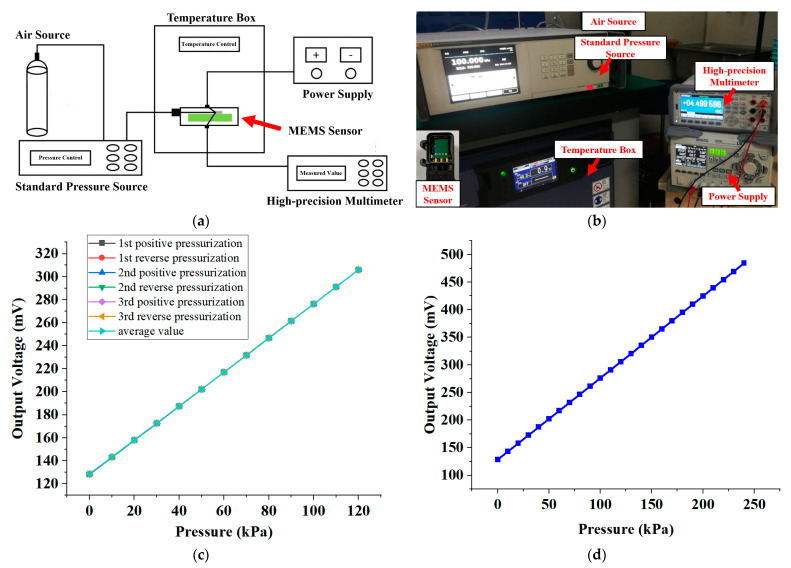
MEMS pressure sensor performance test. (**a**) Schematic diagram of the test experiment system. (**b**) Photographs of test experiment systems and package samples. (**c**) Test results of 3 cycles of pressure calibration of the same sensor over the whole range (0–120 KPa). (**d**) The maximum overvoltage test result of the sensor chip. When the pressure reaches 240 KPa, the output voltage of the chip is 484.379 mV, and the function is verified without abnormality.

First of all, we conducted an examination of the MEMS pulse pressure sensor, and the constructed test configuration is shown in Figure 6a. In the course of the assessment, the posterior pins of the MEMS pressure sensor with a TSV configuration were joined to a customized PCB to establish a bridge circuit for conduction. Subsequently, the welded PCB was made airtight for the purpose of pressure signal appraisal. Furthermore, the excitation power supply, multimeter, pressure source, and other relevant instrumentation were interlinked. The connected test configuration is shown in Figure 6b.

We conducted a pressure calibration for the MEMS pressure sensor at 10 KPa intervals over the entire range (0–120 KPa), yielding a total of 13 pressure calibration points. Additionally, we conducted three pressure cycle calibration tests on the same sensor. Figure 6c displays the results of the three cycle pressure calibration tests performed on the sensor over the entire range. Furthermore, to determine the maximum overvoltage test pressure, we subjected the sensor to a pressure signal of 240 KPa, held it for 1 min, and then unloaded it. The maximum overvoltage refers to the highest pressure that the sensor can withstand without causing permanent changes in performance. The maximum overvoltage test result of the sensor chip showed that when the pressure reached two times the range of 240 KPa, the output voltage of the chip was 484.379 mV. The function verification did not indicate any abnormalities. We calculated the parameters of the MEMS sensor and present them in Table 2.

### 3.2. Static Pressure Test of Sensor Array

**Figure 7:**
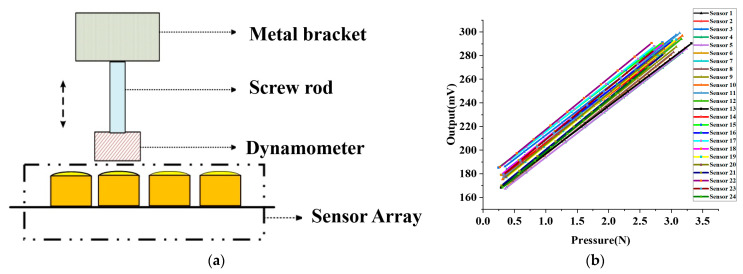
MEMS pressure sensor array static pressure test. (**a**) Schematic diagram of MEMS sensor static pressure test device. (**b**) Linearity calibration of 24 sensors in the sensor array.

Upon completion of the fabrication of the sensor array, which is explained in Section 2.3, we proceeded to test the sensor in its packaged form under static pressure. We designed a calibration device to repeatedly test the packaged sensor under standard conditions. The calibration device, illustrated in Figure 7a, includes a custom metal bracket affixed with a screw capable of vertical movement. When the dynamometer affixed to the screw contacts the packaged sensor, the interaction force between the dynamometer and the sensor is directly measured. The output voltage, V, of the packaged sensor varies with the interaction force F.

As illustrated in Figure 7b, we tested 24 packaged sensors that comprised the array and employed the least squares method to linearly fit the test data to obtain the sensitivity of each sensor. The average sensitivity and standard deviation of the 24 packaged sensors were 42.07 mV/N and 1.1 mV/N, respectively. Our findings reveal that the packaged sensor exhibits a desirable linear relationship between the output voltage and the applied pressure, indicating its excellent linearity that satisfies our pulse wave acquisition requirements. We utilized the linearity calibration results of the sensor for pulse wave acquisition.

To further assess the repeatability of the packaged sensor, which is an indicator of the sensor’s reliability and stability, we randomly selected six packaged sensors and tested each sensor ten times using the same device. We then calculated the repeatability equation as follows:

(4) $${s_{i}}^{2}=\frac{1}{m-1}\sum_{j=1}^{m}{\left(y_{ij}-{\bar{y}}_{i}\right)}^{2}$$

(5) $$s=\sqrt{\frac{1}{2n}\sum_{i=1}^{n}{s_{i}}^{2}}$$

where y is the measured value, $s_{i}$ is the variance of the measurement point i, m represents the number of repetitions of the test experiment (the value is six in this experiment), and n is the number of measurement points with a value of 10. Equations (4) and (5) reveal that the repeatability of the six sensors is 3.4%, 3.1%, 2.4%, 3.7%, 4.2%, and 3.8%, and the average repeatability is 3.4%. The results, which reflect good repeatability in the packaged sensor array, are shown in Table 3.

### 3.3. Dynamic Pulse Test of Sensor Array

**Figure 8:**
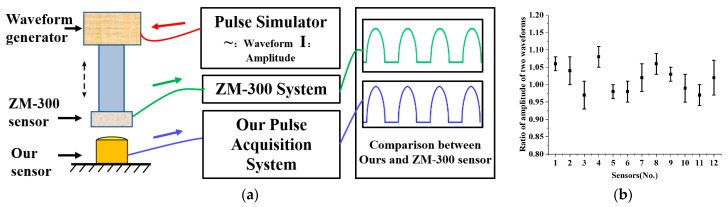
Experiment on dynamic pulse of MEMS pressure sensor array. (**a**) Experimental diagram of sensor dynamic pulse test. (**b**) The amplitude ratio of our designed MEMS sensor to ZM-300.

To evaluate the performance of our packaged sensor when measuring dynamic pulse waves, we conducted a standard experiment to compare it with the ZM-300 pulse sensor, a pulse wave standard acquisition system used as a control. The ZM-300 pulse diagnostic instrument is a classic pulse diagnostic instrument that meets the technical requirements of pulse wave acquisition and is considered to be a pulse wave standard acquisition system that can be used as a control [14,16]. The dynamic pulse wave test experiment setup is illustrated in Figure 8a, with a custom-designed pulse wave waveform generator driving the ZM-300 sensor to simulate human pulse waves. We measured the contact force between the ZM-300 sensor and our sensor synchronously to confirm that the detected pressures were the same. The amplitude ratio of the two pulse wave waveforms was calculated to measure the performance of our sensor array in testing dynamic pulse waves.

We randomly selected twelve sensors for testing and each was tested six times. As is shown in Figure 8b, the average amplitude ratio of the twelve sensors was 1.02, indicating that our MEMS pressure sensor’s dynamic pressure characteristics are comparable to those of the ZM-300 sensor. This result suggests that the sensor array we designed can also be used for dynamic pulse wave acquisition. The standard deviation of linearity of the twelve randomly selected sensors is 3.25%, indicating good consistency between the packaged sensors and demonstrating that the sensor array can be applied to pulse wave acquisition.

### 3.4. Experiment on Human Pulse Wave Acquisition

**Figure 9:**
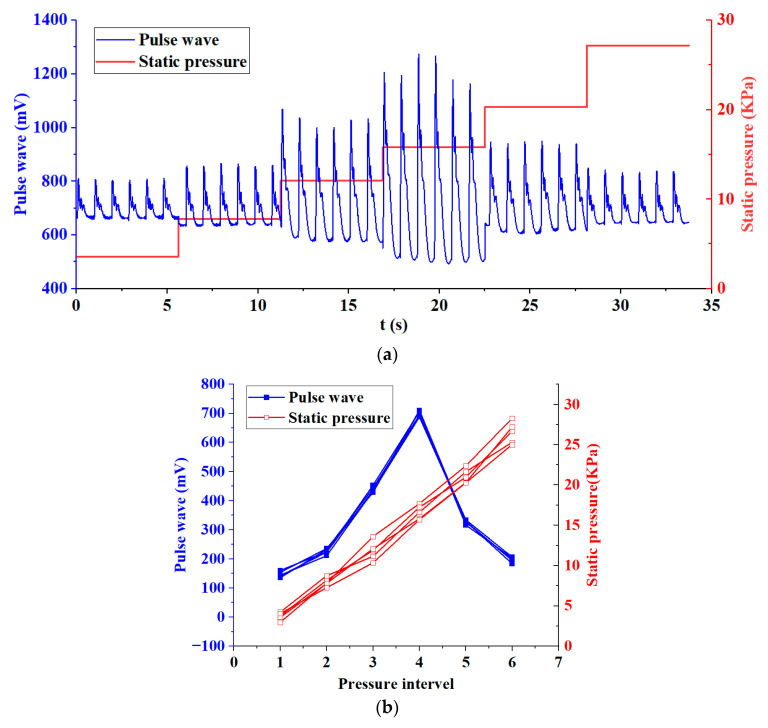
Experiment on human pulse wave acquisition. (**a**) Pulse wave signal and static pressure signal of a volunteer during pulse wave acquisition. (**b**) Pulse wave amplitude and static pressure of a volunteer’s five tests.

In order to evaluate the effectiveness and repeatability of the system for collecting pulse waves on the human body, we conducted five acquisition tests on the wrist of the volunteer. The interval between each collection was three minutes. Six pressure intervals were set for each acquisition. The pressure range was between 0 and 30 KPa. The volunteer was asked to keep his body still and calm during the pressurization period. The pulse wave signal and static pressure signal of this volunteer are shown in Figure 9a. The pulse wave amplitude and static force of the volunteer’s five tests are shown in Figure 9b.

We made statistics on the five test results of the volunteer and calculated the relative standard deviations (RSD) of the static pressure and pulse wave amplitude of the six pressure intervals, as shown in Table 4. It can be seen from Table 4 that only in pressure interval 1, the RSD of static pressure and pulse wave amplitude exceeds 5%. This is because when the pressure applied to the wrist is small, the sensor is not in close contact with the skin, and the interference caused by the volunteer’s slight movement is relatively large, resulting in a higher deviation. In the pressure range 2–6, the RSD are all less than 5%. Especially in pressure interval 4, the pulse wave amplitude reaches the maximum, with less noise and a clearer waveform, which is the interval we focus on. At this time, the RSD of the pulse wave amplitude was 2.39%. This shows that the system has good validity and repeatability when collecting human pulse waves. The pulse wave array we proposed can acquire pulse waves under varying static pressure.

### 3.5. Experiment on Pulse Wave Width Measurement

To verify the feasibility of pulse wave width measurement, we designed an experiment by collecting pulse waves and infrared images of the wrist radial artery. We recruited fifteen volunteers, and their basic information is shown in Table 5. We collected their pulse wave and the infrared image of the corresponding wrist three times, respectively. The interval between collecting the pulse wave and taking the infrared image did not exceed five minutes, and we instructed the subjects not to perform any strenuous activities during the test in order to maintain a calm state of mind and body.

**Figure 10:**
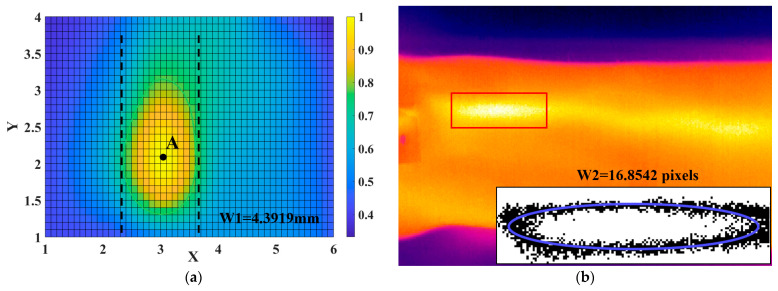
Experiment on pulse wave width measurement. (**a**) The 2D projection plane corresponding to the 3D pulse wave and the width of pulse wave W1. (**b**) The corresponding infrared image and the width of the pulse wave (W2).

At first, according to the algorithm described in Section 2.5, we obtained the 3D pulse wave, and the corresponding two-dimensional projection image is shown in Figure 10a. The pulse wave width was then calculated from the green outline drawn at the threshold, labeled W1. For the infrared image of the wrist radial artery shown in Figure 10b, we calculated the width of the pulse wave in three steps: firstly, we manually located the region of the pulse wave in the image; secondly, after graying the image, we used a threshold to determine the boundary of the region; finally, after fitting with the ellipse equation, the length of the minor axis (that is, the width of the pulse wave) was recorded as W2.

**Figure 11:**
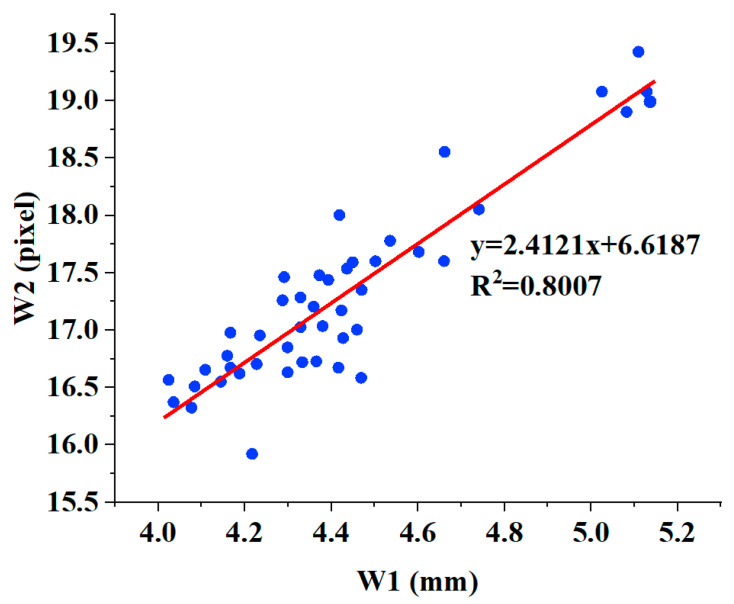
The linear relationship between W2 and W1.

The scatterplots of W1 and W2 are shown in Figure 11. We then performed a linear fit on the data of W1 and W2, and the coefficient $R^{2}$ of determination in linear fit was 0.8007. Due to the limited resolution of the infrared camera, there is a certain error in the width measurement detected using infrared images, resulting in a low fitting correlation coefficient; however, it is still higher than 0.7, which indicates that there is a positive correlation between pulse width W1 and pulse width W2, as detected by the infrared camera. This suggests that the sensor array we designed, combined with the 3D pulse wave algorithm, has the ability to measure pulse width information.

### 3.6. Comparisons with Other Pulse Signal Acquisition Systems

This study proposed a novel wearable multichannel pulse wave acquisition system based on a MEMS pressure sensor flexible array with a TSV structure. The back of the MEMS pressure sensor and the flexible substrate can be directly connected without gold wire bonding, which solves the problem of sensor failure due to the breaking of gold wire, which is common in traditional MEMS pressure sensors. Furthermore, based on the MEMS sensor, we devised a 24-channel pulse pressure sensor flexible array and developed a customized pulse preprocessing chip to process the wrist pulse wave signal. We built an algorithm framework to recover the 3D pulse wave from the pulse wave array signal and calculate the pulse width. Finally, our experimental results verified the effectiveness and reproducibility of the MEMS pressure sensor and array we proposed. In addition, the pulse width calculation result is highly positively correlated with the results obtained via infrared images. This outcome underscores the system’s commendable performance in pulse wave acquisition and analysis, thereby affirming the feasibility of its further clinical application.

A comparison of our proposed system with other pulse wave acquisition analysis systems demonstrated in recent studies is summarized in Table 6. Chen [16], Liu [14], and Kang [13] represent our previous efforts to develop effective pulse wave acquisition devices. Chen [16] proposed a pulse wave signal acquisition system for width information based on a MEMS sensor array, and Kang [13] developed a wearable real-time pulse wave monitoring system based on a flexible compound sensor. Hu [22] implemented a pulse-taking platform with a tactile array sensor and constructed 3D wrist pulse wave signals. Jin [40] built a wearable combined wrist pulse measurement system using airbags for pressurization, and Wang [29] developed a novel 9-channel pulse monitoring platform and made remarkable progress.

Compared with other pulse signal acquisition systems, the proposed system had several advantages. Firstly, the MEMS pressure sensor we proposed uses a TSV structure, which solves the problem of the existing pressure sensor’s wire bonding that is easily broken and causes the sensor to fail and improves the pressure detection range and the service life of the sensor. Secondly, the sensors have been taped out on 8-inch wafers, which can be produced on a large scale to reduce costs. Compared with flexible sensors, the MEMS sensor has a simple structure, a mature technology, good consistency, and linearity. Thirdly, in terms of the number of sensor arrays, compared with existing pulse acquisition systems, our proposed system can use the largest number of sensors because our sensor size is only 2 mm × 2 mm. The array can completely cover the radial artery area and can collect the pulse wave information of the wrist radial artery more precisely, making the measurement of pulse width more accurate. Finally, the pulse wave signal collected by the sensor array can reconstruct a finer 3D pulse wave and provide rich pulse wave information. More pulse information helps doctors make a more accurate diagnosis.

## 4. Conclusions

In conclusion, our system for pulse wave acquisition and analysis is both feasible and effective. The system is based on a novel MEMS pressure sensor with a TSV structure, whose back can be directly connected to a flexible substrate without gold wire bonding. Moreover, a 24-channel pulse pressure sensor flexible array was designed using a MEMS sensor, along with a customized pulse acquisition and processing chip and signal processing algorithm that allow for the recovery of the 3D pulse wave from the pulse wave array and the calculation of the pulse width. Additionally, the small size and custom-designed acquisition and processing chip enable our system to be wearable and portable, demonstrating significant research value and commercial prospects.

In the future, we plan to further optimize and refine the performance of this system by developing higher-density sensor arrays and more accurate algorithm analysis results. We will also conduct more pulse wave data collection experiments and engage in deeper communication with doctors to make the system more standardized and intelligent. Ultimately, these efforts will be crucial to the modernization of pulse wave diagnosis and people’s health monitoring, and we are excited to continue advancing this technology.

## Figures and Tables

**Table 1:** Parameters of multichannel pulse wave acquisition and processing chip.

Variables	Parameters
Number of pulse signal input channels	8
Single chip power consumption	18.7 mW
Amplifier gain	35.9 dB
Sample rate	1 Msps
ADC bits	10 bits
System sampling frequency	256 Hz

**Table 2:** Parameters of MEMS pressure sensors with a TSV structure.

Variables	Parameters
Chip area	2 mm × 2 mm
Sensitivity	177.5398 mV/120 KPa@5 V
Precision	0.3395% FS
Pressure range	1–120 KPa
Maximum pressure	Over 2 × FS
Nonlinear	0.1062%

**Table 3:** Results of six randomly selected sensor repeatability experiments.

Sensor No.	2	7	9	14	18	23	Average Value
Repeatability	3.4%	3.1%	2.4%	3.7%	4.2%	3.8%	3.4%

**Table 4:** Relative standard deviations (RSD) of pulse wave amplitude and static pressure.

Pressure Interval	1	2	3	4	5	6
RSD of Pulse wave amplitude	6.71%	4.24%	2.21%	2.39%	2.06%	4.53%
RSD of Static Pressure	6.25%	3.27%	2.41%	4.46%	3.69%	4.05%

**Table 5:** Basic information of the fifteen volunteers in the pulse width measurement test.

Variables	Parameters
Gender	
Male	8
Female	7
Age (year)	32.1 ± 7.3 (21–46)
Height (cm)	171.6 ± 8.2 (155–190)
Weight (Kg)	62.6 ± 12.8 (41–101)
BMI	21.2 ± 3.1 (17.1–30.2)

**Table 6:** Comparisons with other pulse measurement systems.

System	Hu [22]	Liu [14]	Jin [40]	Chen [16]	Kang [13]	Wang [29]	Proposed
Number of sensors	12	5	3	12	1	9	24
Array	3 × 4	1 × 5	1 × 3	3 × 4	1	3 × 3	4 × 6
Single sensor size (mm)	2.5 × 2.5	30 × 12	5.6 × 5.6	5.5 × 3.6	40 × 10	2 × 2	2 × 2
Flexible	No	Yes	No	Yes	Yes	No	Yes
Wearable	No	No	No	No	Yes	Yes	Yes
3D pulse wave	Yes	No	No	Yes	No	Yes	Yes
Pulse Width	No	No	No	Yes	No	Yes	Yes
Pulse-taking pressure acquisition	Yes	Yes	Yes	Yes	Yes	No	Yes
Measure under continuously changing pressure	Yes	No	No	No	Yes	No	Yes
Year of publication	2017	2018	2019	2020	2022	2022	2023

## Data Availability

The raw/processed data required to reproduce these findings cannot be shared at this time as the data also form part of an ongoing study.

## References

[1] He D., Wang L., Fan X., Yao Y., Geng N., Sun Y., Xu L., Qian W. (2017). A New Mathematical Model of Wrist Pulse Waveforms Characterizes Patients with Cardiovascular Disease—A Pilot Study. Med. Eng. Phys..

[2] Zhang Z., Zhang Y., Yao L., Song H., Kos A. (2018). A Sensor-Based Wrist Pulse Signal Processing and Lung Cancer Recognition. J. Biomed. Inform..

[3] Moura N., Ferreira A. (2016). Pulse Waveform Analysis of Chinese Pulse Images and Its Association with Disability in Hypertension. J. Acupunct. Meridian Stud..

[4] Wang G., Geng X., Kang X., Zhang Y., Zhang J., Zhang H. (2022). A Novel Radial Artery P-S Curve Model Based on Radial Vibration of Vascular Wall. Appl. Sci..

[5] Liu Z.-D., Liu J.-K., Wen B., He Q.-Y., Li Y., Miao F. (2018). Cuffless Blood Pressure Estimation Using Pressure Pulse Wave Signals. Sensors.

[6] Jatoi N., Mahmud A., Bennett K., Feely J. (2009). Assessment of arterial stiffness in hypertension: Comparison of oscillometric (Arteriograph), piezoelectronic (Complior) and tonometric (SphygmoCor) techniques. J. Hypertens..

[7] Zhang Y., Cui J., Ma K., Chen H., Zhang J. (2020). A wristband device for detecting human pulse and motion based on the Internet of Things. Meas. J. Int. Meas. Confed..

[8] Tsai Y., Huang Y., Lin S., Lee S., Cheng Y., Chang Y., Su Y. (2018). Different harmonic characteristics were found at each location on TCM radial pulse diagnosis by spectrum analysis. Evid. Based Complement. Altern. Med..

[9] Fine J., McShane M.J., Coté G.L., Scully C.G. (2022). A Computational Modeling and Simulation Workflow to Investigate the Impact of Patient-Specific and Device Factors on Hemodynamic Measurements from Non-Invasive Photoplethysmography. Biosensors.

[10] Miao F., Wang X., Yin L., Li Y. (2019). A wearable sensor for arterial stiffness monitoring based on machine learning algorithms. IEEE Sens. J..

[11] Chen J., Zhang J., Hu J., Luo N., Sun F., Venkatesan H., Zhao N., Zhang Y. (2022). Ultrafast-Response/Recovery Flexible Piezoresistive Sensors with DNA-Like Double Helix Yarns for Epidermal Pulse Monitoring. Adv. Mater..

[12] Martín-Escudero P., Cabanas A.M., Fuentes-Ferrer M., Galindo-Canales M. (2021). Oxygen Saturation Behavior by Pulse Oximetry in Female Athletes: Breaking Myths. Biosensors.

[13] Kang X., Zhang J., Shao Z., Wang G., Geng X., Zhang Y. (2022). A Wearable and Real-Time Pulse Wave Monitoring System Based on a Flexible Compound Sensor. Biosensors.

[14] Liu S., Zhang S., Zhang Y., Geng X., Zhang J., Zhang H. (2018). A novel flexible pressure sensor array for depth information of radial artery. Sens. Actuators A Phys..

[15] Chen J., Sun K., Zheng R., Sun Y., Yang H., Zhong Y., Li X. (2021). Three-Dimensional Arterial Pulse Signal Acquisition in Time Domain Using Flexible Pressure-Sensor Dense Arrays. Micromachines.

[16] Chen C., Li Z., Zhang Y., Zhang S., Hou J., Zhang H. (2020). A 3D Wrist Pulse Signal Acquisition System for Width Information of Pulse Wave. Sensors.

[17] Selvaraj N., Shelley K., Silverman D., Stachenfeld N., Galante N., Florian J., Mendelson Y., Chon K. (2011). A novel approach using time-frequency analysis of pulse-oximeter data to detect progressive hypovolemia in spontaneously breathing healthy subjects(optical). IEEE Trans. Biomed. Eng..

[18] Chang H., Chen J., Liu Y. (2018). Micro-piezoelectric pulse diagnoser and frequency domain analysis of human pulse signals. J. Tradit. Chin. Med. Sci..

[19] Zhang S., Cao J., Xu L., Wang F., Zhang X., Li G., Fang P., Zhu G. A Piezoelectret-based Flexible Sensor for Pulse Monitoring. Proceedings of the 2018 IEEE International Conference on Cyborg and Bionic Systems (CBS).

[20] Liu L., Zuo W., Zhang D., Li N., Zhang H. (2012). Combination of heterogeneous features for wrist pulse blood flow signal diagnosis via multiple kernel learning. IEEE Trans. Inf. Technol. Biomed..

[21] Zhang D., Zuo W., Zhang D., Zhang H., Li N. (2010). Wrist blood flow signal-based computerized pulse diagnosis using spatial and spectrum features. J. Biomed. Sci. Eng..

[22] Hu C., Chung Y., Yeh C., Luo C. (2012). Temporal and spatial properties of arterial pulsation measurement using pressure sensor array. Evid. Based Complement. Altern. Med..

[23] Jia D., Chao J., Li S., Zhang H., Yan Y., Liu T., Sun Y. (2018). A Fiber Bragg Grating Sensor for Radial Artery Pulse Waveform Measurement. IEEE Trans. Biomed. Eng..

[24] Leitão C., da Costa Antunes P., Bastos J., Pinto J., de Brito André P. (2014). Plastic Optical Fiber Sensor for Noninvasive Arterial Pulse Waveform Monitoring. IEEE Sens. J..

[25] Kim B., Hong Y., An Y., Kim S., Lee H., Kim S., Hong S., Yun G., Yook J. (2016). A Proximity Coupling RF Sensor for Wrist Pulse Detection Based on Injection-Locked PLL. IEEE Trans. Microw. Theory Tech..

[26] Nie B., Li R., Brandt J.D., Pan T. (2014). Iontronic microdroplet array for flexible ultrasensitive tactile sensing. Lab Chip.

[27] Boutry C.M., Nguyen A., Lawal Q.O., Chortos A., Rondeau-Gagné S., Bao Z. (2015). A sensitive and biodegradable pressure sensor array for cardiovascular monitoring. Adv. Mater..

[28] Bai N., Wang L., Wang Q., Deng J., Wang Y., Lu P., Huang J., Li G., Zhang Y., Yang J. (2020). Graded intrafillable architecture-based iontronic pressure sensor with ultra-broad-range high sensitivity. Nat. Commun..

[29] Wang J., Zhu Y., Wu Z., Zhang Y., Lin J., Chen T. (2022). Wearable multichannel pulse condition monitoring system based on flexible pressure sensor arrays. Microsyst. Nanoeng..

[30] Chu Y., Zhong J., Liu H., Ma Y., Liu N., Song Y., Liang J., Shao Z., Sun Y., Dong Y. (2018). Human pulse diagnosis for medical assessments using a wearable piezoelectret sensing system. Adv. Funct. Mater..

[31] Wang B., Liu C., Xiao Y., Zhong J., Li W., Cheng Y., Hu B., Huang L., Zhou J. (2017). Ultrasensitive cellular fluorocarbon piezoelectret pressure sensor for self-powered human physiological monitoring. Nano Energy.

[32] Lin D., Zhang A., Gu J., Chen X., Wang Q., Yang L., Chou Y., Liu G., Wang J. (2018). Detection of multipoint pulse waves and dynamic 3D pulse shape of the radial artery based on binocular vision theory. Comput. Methods Programs Biomed..

[33] Jiao D., Ni Z., Wang J., Li X. (2020). Ultra-small pressure sensors fabricated using a scar-free microhole inter-etch and sealing (MIS) process. J. Micromech. Microeng..

[34] Fei Z.F. (2003). Contemporary Sphygmology in Traditional Chinese Medicine.

[35] Wang S., Zhang Z., Chen Z., Mei D., Wang Y. (2022). Development of Pressure Sensor Based Wearable Pulse Detection Device for Radial Pulse Monitoring. Micromachines.

[36] Andreozzi E., Sabbadini R., Centracchio J., Bifulco P., Irace A., Breglio G., Riccio M. (2022). Multimodal Finger Pulse Wave Sensing: Comparison of Forcecardiography and Photoplethysmography Sensors. Sensors.

[37] Chan M., Ganti V.G., Heller J.A., Abdallah C.A., Etemadi M., Inan O.T. (2021). Enabling Continuous Wearable Reflectance Pulse Oximetry at the Sternum. Biosensors.

[38] Fu Y., Zhao S., Zhu R. (2018). A Wearable Multifunctional Pulse Monitor Using Thermosensation-Based Flexible Sensors. IEEE Trans. Biomed. Eng..

[39] Yuan Y., Liu B., Li H., Li M., Song Y., Wang R., Wang T., Zhang H. (2022). Flexible Wearable Sensors in Medical Monitoring. Biosensors.

[40] Jin C., Xia C., Zhang S., Wang L., Wang Y., Yan H. (2019). A wearable combined wrist pulse measurement system using airbags for pressurization. Sensors.

